# *In Situ* Live Imaging of Gut Microbiota

**DOI:** 10.1128/mSphere.00545-21

**Published:** 2021-09-29

**Authors:** Zhi Zhang, Duo Xu, Jianyang Fang, Dai Wang, Jie Zeng, Xiaodong Liu, Shouqiang Hong, Yunxin Xue, Xianzhong Zhang, Xilin Zhao

**Affiliations:** a State Key Laboratory of Molecular Vaccinology and Molecular Diagnostics, School of Public Health, Xiamen University, Xiamen, Fujian, China; b Center for Molecular Imaging and Translational Medicine, School of Public Health, Xiamen University, Xiamen, China; c Public Health Research Institute, New Jersey Medical School, Rutgers University, Newark, New Jersey, USA; d Department of Microbiology, Biochemistry & Molecular Genetics, New Jersey Medical School, Rutgers University, Newark, New Jersey, USA; University of Michigan-Ann Arbor

**Keywords:** gut microbiota, noninvasive imaging of gut microbiota, ^18^F-FDS, nuclide-labeled small molecules, spatial-temporal distribution, antimicrobial-mediated gut microbiota depletion, fecal microbiota transplantation

## Abstract

Most studies of gut microbiota have focused on relationships between a specific disease and the presence/abundance of one or a few bacterial species/genera. Whether the spatial and temporal distribution of gut microbiota, as a whole, affects or correlates with health is unknown, largely due to the absence of tools for dynamically monitoring the overall gut microbiota landscape inside living subjects. Here, we describe a novel, noninvasive, live imaging method for gut microbiota using 2-deoxy-2-[^18^F]fluoro-d-sorbitol (^18^F-FDS), a compound that specifically labeled gut bacteria in mice and hamsters following oral administration. Positron emission tomography-computed tomography (PET-CT) scanning showed that the radiolabel signal was concentrated in the gut (especially the large intestine), was absent when mice gut microbiota was depleted by antibiotic treatment, and was restored after transplanting antibiotic-treated mice with a fecal or probiotic bacterial mixture. Thus, ^18^F-FDS images microbiota, not gut tissue. The tissue distribution of ^18^F-FDS was the highest in the gut (∼3-fold higher than average), in contrast to 2-deoxy-2-[^18^F]fluoro-d-glucose, which concentrated in brain and many other organs. 2-[^18^F]fluoro-aminobenzoic acid, another bacterium-specific radioactive tracer, was unsuited for gut microbiota imaging due to unexpected stomach retention following oral administration. When similar gut microbiota imaging was done with hamsters, the spatial resolution increased significantly over that with mice, suggesting that even higher spatial resolution can be achieved with humans or large animals. Thus, our work establishes a new tool for noninvasive, live imaging of gut microbiota; the new tool may enable exploration of relationships between gut microbiota landscape and diseases in clinical settings.

**IMPORTANCE** Gut microbiota dysbiosis correlates with many diseases, but such correlations derive mostly from relationships between one or a few bacteria and a particular disease. Since microbiota resemble complex forest ecosystems more closely than individual patches of trees, the overall landscape (spatial and temporal distribution) of gut bacteria may also affect/reflect disease development. Such a possibility has not been explored due to a lack of tools for directly visualizing natural landscape patterns of gut microbiota. The present work identified 2-deoxy-2-[^18^F]fluoro-d-sorbitol as a gut microbiota-specific radioactive tracer and developed a novel PET-CT scan-based imaging method that enables noninvasive, real-time imaging of the overall gut bacterial landscape. The method showed increased spatial resolution when hamsters replaced mice, suggesting that even higher spatial resolution could be achieved with larger animals such as humans. This novel technology establishes the feasibility of investigating spatial-temporal distribution dynamics of gut microbiota with many human diseases.

## INTRODUCTION

Many microbes live in and on humans. Some are commensals, and some are pathogens. The normal gut microbiota, which contains more cells than the host (1, 2), is comprised of hundreds of bacterial species (3–5). These bacteria affect a variety of host functions that include the immune system, nutrient assimilation, metabolism, cell proliferation, intestinal homeostasis, and a variety of diseases (6, 7). In a sense, the human body can be considered to be a “superorganism” (8), since the behavior of gut microbiota cannot be separated from the influence of host behavior nor can host function be separated from features of gut microbiota.

Gut microbiota are generally studied by culturing the microbes, by 16S rRNA-encoding gene sequence analysis, and by genomic deep sequencing of fecal samples or gut dissection products (9–11). Conclusions from analyses of individual components are limited by the inability to culture much of the gut microbiota (12). Moreover, it is difficult to ensure that the proportion of the original components in samples does not change during culturing. Furthermore, the composition of fecal microbiota, which is easy to access, is very different from that of natural gut microbiota (12). Even within different parts of the digestive tract, the microbial composition varies from niche to niche (7, 12). Such differences cannot be revealed using fecal samples.

Recent work shows that the distribution/colonization of bacteria in the intestinal tract can be studied by fluorescent labeling of a limited number of bacterial species through genetic/chemical engineering and transplantation into sterile mice by gastric or rectal administration (13–15). However, this approach requires sacrifice of experimental animals, precluding the study of the natural spatial and temporal organization of gut microbiota in real time within the same animal before and after experimental manipulation. Moreover, current fluorescent labeling methods are suitable only for a fraction of gut bacteria, because the intestinal tract contains a large number of nonculturable microorganisms (12) and because only a few gut bacterial species can be genetically engineered for fluorescence labeling. In addition, the time required for colonization to reach the natural steady-state situation is unknown, and surgical sampling may destroy the natural, three-dimensional distribution of bacterial species within gut microbiota. Even when *in situ* labeling is achieved with fluorescent dyes in the natural niche of indigenous species, penetration of fluorescent light is usually too weak to be expanded from small-animal experiments into human clinical studies and diagnostics (15–17). Thus, novel technologies are needed for *in situ* imaging of gut microbiota in living subjects involving little perturbation of the native microbiota ecology and using probe signals that are strong enough to allow noninvasive recording of gut microbiota landscapes in large experimental animals and humans.

Since the study of gut microbiota is an ecological problem, the spatial organization of gut microbiota has a vital role in microbial succession, community stability, syntrophic relationships, and resiliency (13, 18). Current methods allow the study only of a very limited number of individual bacterial species, and they rely on information collected from only a few gut areas when deducing the overall ecology of the gut microbiota of an animal. Due to the lack of research methods, little is known about either the overall distribution of species within gut microbiota or its effect on/correlation with diseases. Being able to image gut microbiota as whole units would constitute a way to learn about gross, real-time changes in the distribution pattern of gut microbiota ecological landscape in response to controlled perturbations or disease conditions.

Imaging of cancer cells identified 2-deoxy-2-[^18^F]fluoro-d-glucose (^18^F-FDG) as useful in the diagnosis of tumors due to its localization in rapidly metabolizing cells; 2-deoxy-2-[^18^F]fluoro-d-sorbitol (^18^F-FDS) has been used to image brain diseases (19). Moreover, ^18^F-FDS is preferentially absorbed by Gram-negative bacteria, especially *Enterobacteriaceae* (20). That allows diagnostic imaging of some infections. Another radiolabeled compound, 2-[^18^F]fluoro-*para*-aminobenzoic acid (2-^18^F-PABA) or [^11^C]PABA, has been reported to be preferentially adsorbed and retained by both Gram-positive and Gram-negative bacteria (Staphylococcus aureus and Escherichia coli) (21, 22). This probe has been used successfully for distinguishing infection from inflammation. One or more of these compounds may be applicable for *in situ* imaging of overall gut microbiota due to the ability to label many bacteria specifically and simultaneously and due to the strong penetration ability of radioactive signals. Such compounds have not been examined for microbiota imaging.

In the present work, we synthesized ^18^F-FDS and ^18^F-PABA, which we administered orally to mice and hamsters for noninvasive macroscopic stereoscopic imaging. ^18^F-FDS proved to be suitable for such imaging, while ^18^F-PABA and ^18^F-FDG were not due to stomach retardation and specificity issues. ^18^F-FDS-based positron emission tomography-computed tomography (PET-CT) scanning selectively imaged gut bacteria with spatial resolution being improved as animal size increased. The method provides a new way to study the role of intestinal microecology in disease, and it potentially provides a new molecular imaging tool for prospective diagnosis of gut microbiota-related diseases in humans.

## RESULTS

### Preparation and characterization of ^18^F-FDS and 2-^18^F-PABA radiotracers.

^18^F-FDS was prepared as previously described (19) with slight modification (see Fig. S1A in the supplemental material). Radio-TLC (thin-layer chromatography) analysis showed ^18^F-FDS migrating as a single peak, indicating high purity of the reaction product (Fig. S1B). The *R_f_* values for ^18^F-FDS and ^18^F-FDG (used as a substrate for ^18^F-FDS synthesis) were 0.99 and 1.24, respectively (Fig. S1B). When we simulated the acidic gastric environment by incubating the radioactive samples for 1 h at 37°C in hydrochloric acid (pH 1), ^18^F-FDS exhibited a single peak at the same position as that of untreated ^18^F-FDS in radio-TLC analysis (Fig. S1C). Thus, ^18^F-FDS is stable under acidic conditions, as required for oral administration.

10.1128/mSphere.00545-21.2 FIG S1 Synthesis and characterization of 2-deoxy-2-[^18^F]fluoro-d-sorbitol (^18^F-FDS). (A) Schematic diagram of synthesis reaction. (B) Separation of 2-deoxy-2-[^18^F]fluoro-d-glucose (^18^F-FDG) and ^18^F-FDS by thin-layer chromatography (TLC). TLC was performed with CH_3_CN:H_2_O (80:20). Shown are migration of ^18^F-FDG (top, *R_f_* = 1.24), ^18^F-FDS (middle, *R_f_* = 0.99), and an equal mixture of ^18^F-FDG and ^18^F-FDS (bottom). Red line represents resolution time interval of each peak that can be used to calculate area of the peak. (C) TLC results show effects of ^18^F-FDS treatment (top) or no treatment (bottom) with HCl (pH 1) for 1 h at 37°C. *x* axis indicates resolution time; *y* axis indicates radioactivity signal expressed as instrument voltage output. Download FIG S1, TIF file, 0.6 MB. Copyright © 2021 Zhang et al. 2021 Zhang et al. https://creativecommons.org/licenses/by/4.0/ This content is distributed under the terms of the Creative Commons Attribution 4.0 International license.

We developed a new approach for synthesis of 2-^18^F-PABA (Fig. S2A and Text S1). High-performance liquid chromatography (HPLC) analysis showed 2-^18^F-PABA as a single peak at 7.76 min; nonradioactive PABA migrated at 7.65 min (Fig. S2B), indicating successful synthesis of 2-^18^F-PABA. The total radiochemical synthesis time was 40 to 50 min; overall decay-corrected radiochemical yield was 41% ± 4% (*n* = 5); radiochemical purity was >99% (after HPLC purification). The specific activity of 2-^18^F-PABA was 66 ± 18 GBq/μmol. This probe remained stable in saline, serum, or an acidic environment for 2 h after preparation (Fig. S2C to E).

10.1128/mSphere.00545-21.1 TEXT S1 1. Synthesis and characterization of 2-[^18^F]fluoro-*para*-aminobenzoic acid (2-^18^F-PABA). 2. Optimization of antimicrobial treatment regimens for depletion of murine gut microbiota. 3. Reference. Download Text S1, DOCX file, 0.03 MB. Copyright © 2021 Zhang et al. 2021 Zhang et al. https://creativecommons.org/licenses/by/4.0/ This content is distributed under the terms of the Creative Commons Attribution 4.0 International license.

10.1128/mSphere.00545-21.3 FIG S2 Synthesis and characterization of 2-[^18^F]fluoro-*para*-aminobenzoic acid (2-^18^F-PABA). (A) Schematic diagram of synthesis reactions. (B to E) Results for radioactivity HPLC (radio-HPLC) and UV adsorption HPLC (UV-HPLC) detection are shown for an equal mixture of 2-^18^F-PABA and 2-F-PABA (B) and radioactivity-HPLC analysis of 2-^18^F-PABA stability in saline (C), serum (D), or HCl (E) (pH 1) at 37°C for the indicated incubation times. *x* axis indicates resolution time; *y* axis indicates normalized radioactivity signal output as in Fig. S1C. Download FIG S2, TIF file, 0.8 MB. Copyright © 2021 Zhang et al. 2021 Zhang et al. https://creativecommons.org/licenses/by/4.0/ This content is distributed under the terms of the Creative Commons Attribution 4.0 International license.

### PET-CT imaging of gut microbiota.

**FIG 1:**
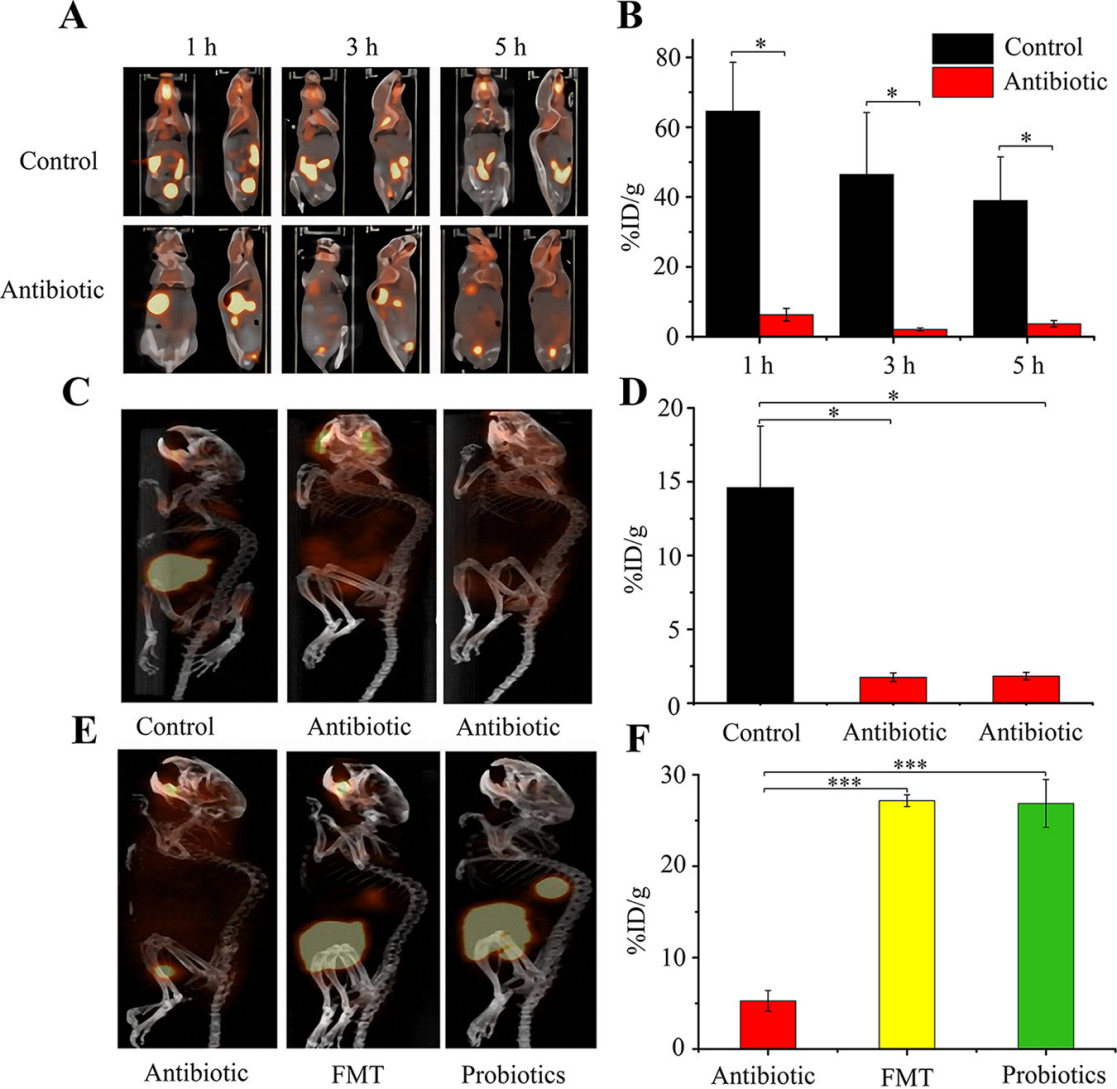
Imaging of gut microbiota following oral administration of ^18^F-FDS. (A) Images of gut microbiota at different times after intragastric feeding of control and clindamycin-plus-ciprofloxacin (Antibiotic)-treated mice with 200 μCi/mouse ^18^F-FDS (*n* = 3). (B, D, and F) Quantification of gut microbiota uptake of ^18^F-FDS derived from PET-CT imaging of panels A, C, and E, respectively. %ID/g is the percentage of injected (input) radioactivity dose per gram of tissue. (C) PET-CT imaging of clindamycin-plus-ciprofloxacin-treated (Antibiotic) and untreated (Control) mice 4 h after ^18^F-FDS administration. Similar results were obtained for three replicate experiments. (E) PET-CT imaging of mice from panel C after microbiota depletion or restoration. The control group mice in panel C were treated with the two antibiotics (left) as in panel C, and the antibiotic-treated groups of mice in panel C were subjected to fecal microbiota transplantation (FMT, middle) or probiotic feeding (right) for a week. Then, mice were administered another dose of ^18^F-FDS by oral gavage, as in panel A. PET-CT imaging was performed 4 h after oral gavage. Representative images, selected from 3 animals per sample point, are shown. *, *P* < 0.05; ***, *P* < 0.001.

To examine the feasibility of *in situ*, noninvasive PET-CT imaging of living mouse gut microbiota with ^18^F-FDS or 2-^18^F-PABA, mice received 200 μCi (in 200 μl saline) ^18^F-FDS or 2-^18^F-PABA by oral gavage before or after depletion of gut microbiota with ciprofloxacin plus clindamycin by oral gavage (this antibiotic combination was chosen from 3 tested regimens because it caused few adverse effects [see Text S1 and Fig. S3). PET-CT scans showed that the ^18^F-FDS signal preferentially localized in gut bacteria (Fig. 1A to D), with the intestinal uptake (percent injected dose per gram of tissue [%ID/g]) of ^18^F-FDS in untreated mice being 6 to 9 times higher than in antibiotic-treated mice (Fig. 1B and D). Moreover, after we reconstructed gut microbiota of antibiotic-treated animals by feeding healthy mouse fecal microbiota or a commercial probiotic bacterial mixture, the intestinal uptake (%ID/g) demonstrated a remarkable increase in radioactive signal (Fig. 1C to F). These results indicate that ^18^F-FDS can serve for *in situ*, noninvasive, real-time imaging of gut microbiota: the intestinal tract signal derives from the uptake of ^18^F-FDS by gut microbiota rather than by gut tissue.

10.1128/mSphere.00545-21.4 FIG S3 Effect of antibiotic treatment regimen on total culturable fecal bacterial numbers. Mice (4 per group) were either untreated (Control) or treated with regimen *a* (Drinking: free drinking of water containing a combination of 1 g/liter of ampicillin, neomycin, and metronidazole and 0.5 g/liter vancomycin), *b* (p.o.: oral gavage of 250 mg/kg body weight of ciprofloxacin and clindamycin twice daily), or *c* (Drinking + p.o. = the combination of regimens *a* and *b*). Fecal samples were collected before and every 24 h after antimicrobial treatment. Fecal homogenate was prepared, serially diluted, and plated on LB agar for incubation at 37°C under aerobic (A and C) or anaerobic (B and D) conditions at 37°C. Enumeration of microorganisms was performed after 36 to 48 h of incubation. Panels A and B are for comparison of 3 treatment regimens using the average from pooled samples for each time point. Panels C and D are comparison between control and the oral gavage group with fecal samples being obtained from each individual mouse (at least 3 of 4 mice produced fecal samples for each time point). Colony counts, normalized per gram of feces, are expressed as a function of sampling time. (E) Effect of antibiotic treatment regimens on mouse body weight (*n* = 3 to 4). Mice were treated as above, and their body weight was measured daily. Percent body weight change was calculated using body weight at the time of antimicrobial administration as 100% for the indicated treatment times. **, *P* < 0.01; ***, *P* < 0.001. Download FIG S3, TIF file, 0.3 MB. Copyright © 2021 Zhang et al. 2021 Zhang et al. https://creativecommons.org/licenses/by/4.0/ This content is distributed under the terms of the Creative Commons Attribution 4.0 International license.

Because ^18^F-FDS exhibits preference for Gram-negative bacteria, especially *Enterobacteriaceae* (20), gut microbiota imaging by ^18^F-FDS may underestimate the contribution of Gram-positive bacteria. In an attempt to perform imaging to cover both Gram-negative and Gram-positive organisms, we prepared 2-^18^F-PABA (PABA is reportedly taken up and retained effectively by both Gram-positive and Gram-negative bacteria [22, 23]). *In vitro* characterization did show some balanced uptake and retention of 2-^18^F-PABA between Gram-positive and Gram-negative bacteria (see Text S1 and Fig. S4 for details). However, when *in vivo* PET-CT imaging of gut microbiota was performed using 2-^18^F-PABA, the imaging showed that most of the 2-^18^F-PABA signal was retained in the stomach, regardless of antibiotic-mediated microbiota depletion (Fig. S4C). These data suggest that much of the 2-^18^F-PABA is adsorbed/retained by the stomach wall, thereby failing to enter the intestinal tract, as required for gut microbiota imaging. Since previous work using intravenous administration of ^18^F-PABA for imaging bacterial infection also showed little signal in the gut (21), 2-^18^F-PABA is unlikely to be an effective gut microbiota-specific imaging agent. It was not examined further.

10.1128/mSphere.00545-21.5 FIG S4 *In vitro* labeling of bacteria and *in vivo* gut microbiota imaging using 2-^18^F-PABA. (A) *In vitro* uptake and retention of 2-^18^F-PABA by E. coli and S. aureus. Bacteria were grown to exponential phase and then labeled with 2-^18^F-PABA at a concentration of 5 μCi (185 kBq)/ml for the indicated times. Radioactivity was determined after cells were pelleted by centrifugation. As a negative control, heat-killed (HK) bacteria were labeled for 90 min and processed as above. To measure 2-^18^F-PABA retention, bacteria were first incubated with 2-^18^F-PABA for 90 min. Then, cells were collected by centrifugation, washed with fresh medium to remove cell-free 2-^18^F-PABA, incubated in fresh medium for another 120 min at 37°C, and then pelleted and measured for radioactivity (90R120: 90-min label followed by 120-min postlabel incubation). **, *P* < 0.01; ***, *P* < 0.001. (B) Competition with nonradioactive 2-^19^F-PABA for *in vitro* uptake of 2-^18^F-PABA with E. coli and S. aureus. Bacteria were grown to exponential phase, after which 2-^18^F-PABA was added to a final concentration of 2 μCi (74 KBq)/ml. At the same time, 2-^19^F-PABA was added at the indicated concentrations, and incubation was continued for 120 min before cells were concentrated and radioactivity was determined as in panel A. (C) PET-CT imaging of murine microbiota. Control and antibiotic (ciprofloxacin plus clindamycin)-treated mice were treated with 200 μCi 2-^18^F-PABA per mouse by oral gavage. Imaging was performed 4 h after radiotracer administration. Download FIG S4, TIF file, 1.0 MB. Copyright © 2021 Zhang et al. 2021 Zhang et al. https://creativecommons.org/licenses/by/4.0/ This content is distributed under the terms of the Creative Commons Attribution 4.0 International license.

### *Ex vivo* quantification of gut microbiota.

**FIG 2:**
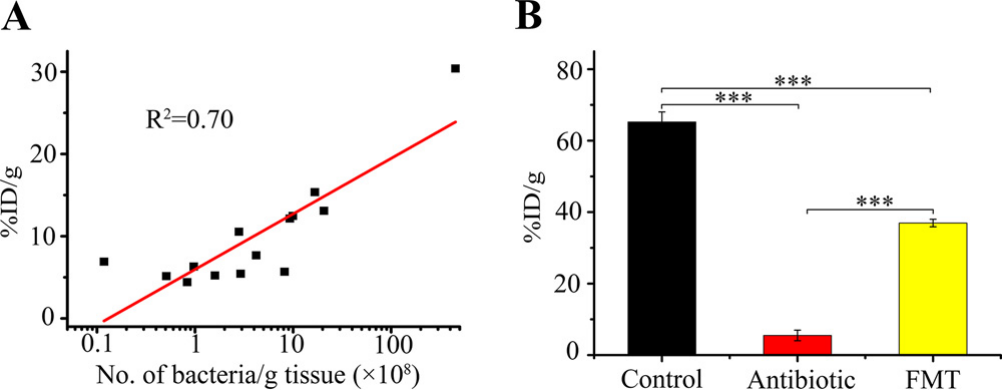
Correlation of gut bacteria with ^18^F-FDS radioactivity. (A) Radioactivity intensity in segmented mouse intestine correlates with Gram-stained bacterial count. Intestines of ^18^F-FDS-treated mice were cut into 2- to 3-centimeter segments. Radioactivity of each segment, expressed as percentage of injected radioactivity dose per gram of tissue (%ID/g), was measured with a gamma counter, and then a homogenate from each segment was subjected to Gram staining and microscopy for bacterial counts. (B) Comparison of radioactivity intensities in cecum from control, antibiotic-treated (ciprofloxacin plus clindamycin), and antibiotic-treated but fecal-microbiota-transplanted mice (FMT). Mice were untreated, treated with antibiotics (ciprofloxacin plus clindamycin), or treated with antibiotics but followed by fecal microbiota transplantation after which ^18^F-FDS was administered for 4 h (*n* = 3). Then, cecum samples were dissected and homogenized, and radioactivity was measured with a gamma counter. ***, *P* < 0.001.

To confirm that the radioactive sorbitol signal arose from uptake by gut microbiota, we surgically removed the entire intestinal tract of mice after imaging, sectioned it, and measured the radioactivity in each segment by γ-counting. We then performed microscopy following Gram staining of bacterial samples recovered from each sectioned gut segment. A good correlation was observed between radioactivity and the number of bacteria in the intestinal samples (*R*^2^ = 0.70) (Fig. 2A).

Since the number of bacteria in the cecum is 10 to 100 times higher than in other intestinal segments (11), we focused on cecum radioactivity after treating mice in several ways. The radioactivity intensity in cecum tissue decreased significantly (∼12-fold) after antimicrobial-mediated depletion of gut microbiota, and it recovered after fecal microbiota transplantation (Fig. 2B). These results show that the radioactive signal correlates quantitatively with bacterial load in the gut and further support the feasibility of *in situ*, noninvasive imaging of gut microbiota using ^18^F-FDS.

### Comparison of imaging by ^18^F-FDS (sorbitol) and ^18^F-FDG (glucose).

**FIG 3:**
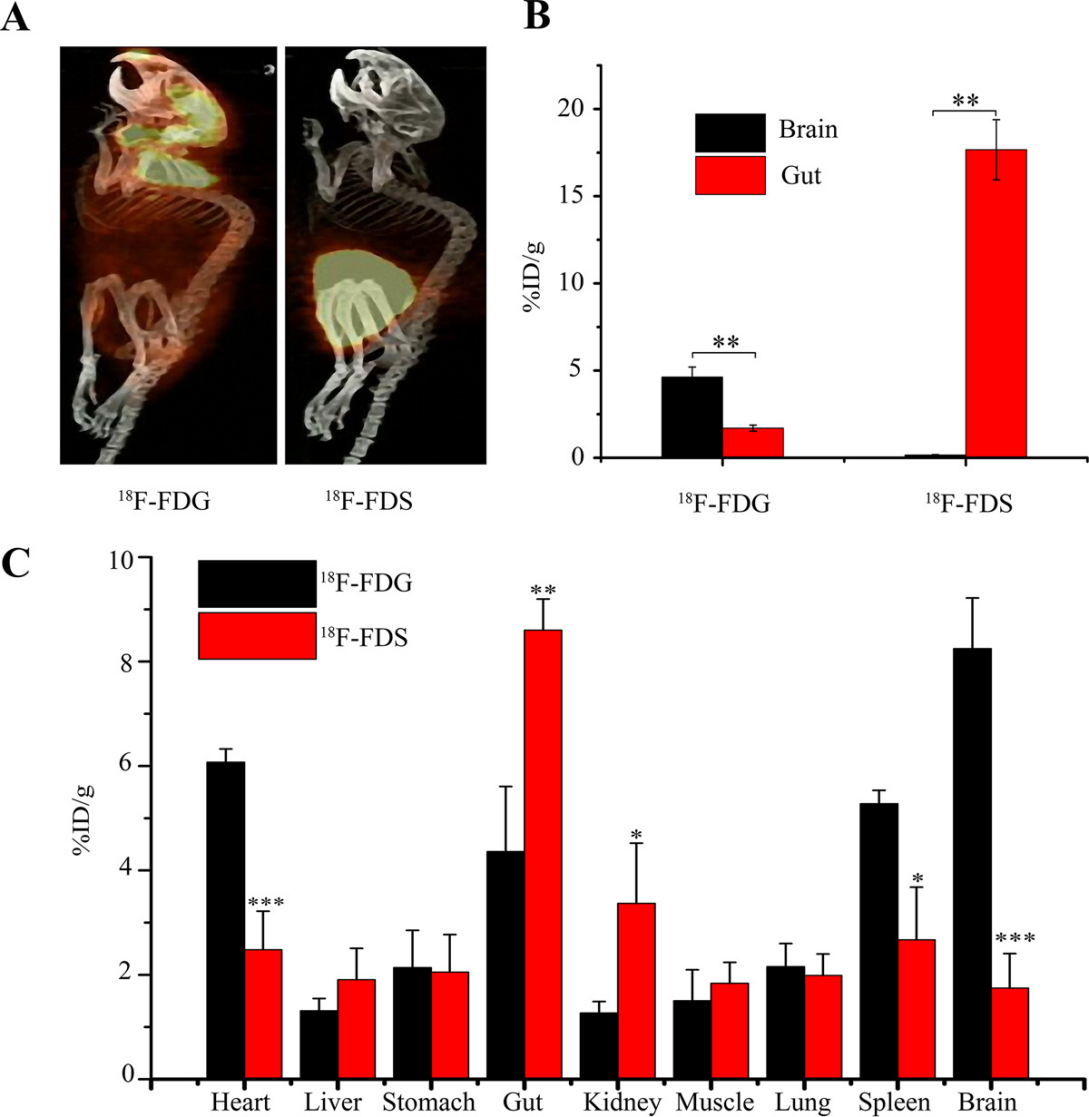
Comparison of distribution of ^18^F-FDG and ^18^F-FDS in mice. (A) PET-CT imaging was performed with mice 4 h after gastric gavage using 200 μCi/mouse ^18^F-FDG or ^18^F-FDS. Images use the same scale for all animals; similar results were observed with three mice. (B) Quantification of gut microbiota and brain uptake of ^18^F-FDG and ^18^F-FDS by selection of regions of interest of the PET-CT image in panel A followed by calculation and conversion of the relative uptake signal to percentage of injected radioactivity per gram of tissue (%ID/g). (C) Biodistribution of ^18^F-FDG and ^18^F-FDS in various organs/tissues. Error bars indicate standard error of the mean. *, *P* < 0.05; **, *P* < 0.01; ***, *P* < 0.001.

^18^F-FDG is an FDA-approved, commercially available radiotracer widely used for clinical PET-CT scans. If this compound is also suitable for imaging gut microbiota, it might be quickly approved for clinical applications with humans. Consequently, we compared imaging of gut microbiota with PET-CT scanning performed 4 h after oral administration of ^18^F-FDS or ^18^F-FDG to mice. ^18^F-FDG failed to localize to the gut, although it was enriched in brain and heart, along with a general distribution over the entire body. In contrast, the radioactive signal for ^18^F-FDS was predominantly in the gut, with very low background in other body parts (Fig. 3A and B). We also used gamma counting to examine the biodistribution of ^18^F-FDG and ^18^F-FDS following surgical extraction of various organs and tissues after imaging. ^18^F-FDG tended to localize in the heart, brain, and spleen, while ^18^F-FDS preferentially localized in the intestine (Fig. 3C). These data support the conclusion from PET-CT imaging that ^18^F-FDS, but not ^18^F-FDG, is suitable for imaging gut microbiota.

### ^18^F-FDS imaging in hamster.

**FIG 4:**
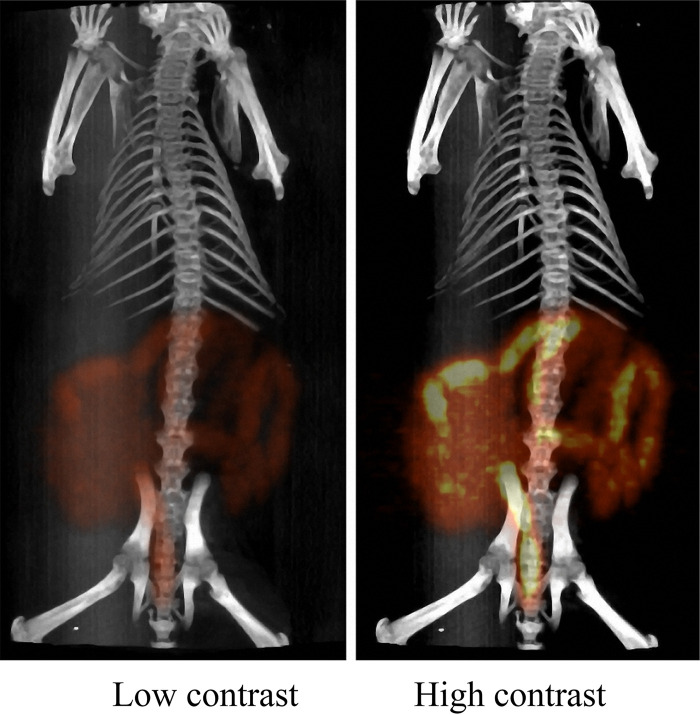
Imaging of gut microbiota of Syrian hamster with ^18^F-FDS. Syrian hamsters were administered 400 μCi/animal ^18^F-FDS by oral gavage; PET-CT scan image was taken 6 h later. The low-contrast image emphasizes that the large intestine was preferentially labeled; the high-contrast image shows that the entire intestine is labeled, with the large intestine exhibiting the highest signal. Similar results were obtained from 3 animals.

Since mice are small, the gut imaging signal is condensed in the intestine area, making finer spatial distribution difficult to observe. To explore whether spatial resolution increases with body size, we next examined imaging with hamster, the largest animal that our instrument could accommodate. The spatial resolution with Syrian hamster (Fig. 4 and Movie S1) was much higher than observed with mouse (Fig. 1C and Movie S2). Thus, an increase in body size drastically enhances the spatial resolution of gut microbiota imaging with ^18^F-FDS. This apparent body-size effect increases the feasibility for obtaining high-resolution imaging of the overall gut microbiota landscape in humans or in large-animal disease models.

10.1128/mSphere.00545-21.7 MOVIE S1 Rotation of 3-dimensional imaging of hamster gut microbiota by ^18^F-FDS. Download Movie S1, MOV file, 0.9 MB. Copyright © 2021 Zhang et al. 2021 Zhang et al. https://creativecommons.org/licenses/by/4.0/ This content is distributed under the terms of the Creative Commons Attribution 4.0 International license.

10.1128/mSphere.00545-21.8 MOVIE S2 Rotation of 3-dimensional imaging of mouse gut microbiota by ^18^F-FDS. Download Movie S2, MOV file, 0.9 MB. Copyright © 2021 Zhang et al. 2021 Zhang et al. https://creativecommons.org/licenses/by/4.0/ This content is distributed under the terms of the Creative Commons Attribution 4.0 International license.

## DISCUSSION

The organisms of the gut microbiota influence microbial succession, community stability, and syntrophic relationships between the community and its host intestinal environment (4, 13). Previous work details the spatial organization and distribution of a few bacterial species and classes in the intestinal tract (13–15). However, the number of species requiring such analysis is enormous if we are to understand gut microbiota biology as a whole. Moreover, their interactions are complex, especially those that contribute to the stability of symbiotic relationships among members of the microbial community. Our inability to genetically label most gut bacterial species limits the study of the overall microbiota landscape. Furthermore, either transplantation of *in vitro*-labeled bacteria may disturb the natural ecological landscape or the transplanted bacteria may take too long to settle into their natural niches. The intrinsically weak penetration problem of all fluorescence-based imaging methods makes most, if not all, currently established methods unsuitable as potential noninvasive diagnostic tools for human diseases.

The present work describes a way to study gut microbiota as a unit. The central idea is that a radioactive isotope having enough energy for external detection is incorporated into a carbohydrate that is readily incorporated by diverse gut bacterial species but not by host cells. Sorbitol was superior to glucose in terms of selectivity; it was also superior to *p*-aminobenzoic acid (PABA), a compound that was expected to provide a more balanced uptake by both Gram-negative and Gram-positive gut bacteria. However, PABA failed to meet expectations, because it was sequestered in the stomach following oral administration. Thus, ^18^F-FDS constitutes a lead compound for *in situ*, live imaging of the overall gut microbiota in the same animal before and after experimental perturbation. Follow-up studies in humans and large animals are expected to facilitate many studies of potential links between the overall gut microbiota landscape patterns and disease status.

A key feature of the ^18^F-FDS imaging method is its ability to visualize many bacteria in the natural gut environment without prelabeling *in vitro*, as is not the case with previous fluorescent labeling methods (13–15). Moreover, labeling gut bacteria with fluorescent proteins or cell wall precursors is currently successful with only a limited number of bacterial species, because such proteins usually do not fluoresce in the largely anaerobic environment of the gut and because many gut bacteria are difficult to culture and manipulate genetically. In addition, the few bacterial species that can be labeled and delivered into the gut may not be in their natural niche during the observation period, because colonization and rebalance take time and because it is difficult to conclude that a reconstituted bacterial community represents the fully established natural bacterial community. Furthermore, penetration of fluorescent light is weak, making fluorescence-based imaging applicable only to small animals or to dissected guts that may lose their natural spatial patterns. Fluorescence *in situ* hybridization can cover more bacterial species (13), but it requires dissection and tissue processing, which may also destroy the natural microbiota landscape. Photoacoustic imaging (PAI) is another approach for imaging gut microbiota (24). It is a noninvasive, nonionizing biomedical imaging method. As with fluorescence labeling, signal penetration is weak, making PAI suitable only for dissected guts or small animals. Thus, ^18^F-FDS-based PET-CT scan provides the first platform for probing the natural spatial distribution patterns of gut bacterial communities in living hosts of any size.

We stress that ^18^F-FDS has a preference for labeling Gram-negative relative to Gram-positive bacteria. However, since gut microbiota contain many Gram-negative bacteria and since ^18^F-FDS does label Gram-positive bacteria, although at a lower efficiency, the potential underestimation of the Gram-positive bacterial signal during ^18^F-FDS imaging may have little effect on the overall interpretation of the data. Indeed, the ^18^F-FDS-labeling of an anaerobic Gram-positive gut bacterium, Clostridium difficile, was 3.4-fold better than S. aureus, a representative non-gut-inhabitant, Gram-positive bacterium (see Fig. S5 in the supplemental material). Moreover, feeding a Gram-positive probiotic bacterial mixture successfully restored the ^18^F-FDS signal for guts of mice after their natural gut microbiota were depleted by antibiotic treatment (Fig. 1E and F). These data suggest that the thinner cell wall of anaerobic Gram-positive gut bacteria may help improve ^18^F-FDS labeling. Compounds judged by *in vitro* experiments to better image both Gram-positive and Gram-negative bacteria must be demonstrated for suitability in live hosts for gut microbiota imaging, since orally administered tracers must readily reach the gut without being sequestered elsewhere. PABA represents a cautionary case, as stomach sequestration of the compound makes it unsuitable for gut microbiota imaging.

10.1128/mSphere.00545-21.6 FIG S5 *In vitro* uptake of ^18^F-FDS in E. coli (BW25113), S. aureus (RN450), and C. difficile (VPI 10463). Exponentially growing cultures of bacteria were incubated with 2 μCi/ml of ^18^F-FDS for 120 min. Bacteria were recovered by centrifugation, and free ^18^F-FDS was removed by washing the bacterial pellet 3 times with fresh medium. Radioactivity was determined using a gamma counter, and normalized cellular uptake was expressed as percentage of the amount retained by E. coli. *, *P* < 0.05; ***, *P* < 0.001. Download FIG S5, TIF file, 0.3 MB. Copyright © 2021 Zhang et al. 2021 Zhang et al. https://creativecommons.org/licenses/by/4.0/ This content is distributed under the terms of the Creative Commons Attribution 4.0 International license.

Several features make the ^18^F-FDS-based imaging method readily suitable for clinical applications. First, ^18^F-FDS can directly label bacteria in their natural intestinal niche. Such a feature eliminates reliance on prelabeling and administration of a few *in vitro*-cultured bacterial species to draw conclusions about the bacterial community as a whole. Indeed, the *in situ* labeling of bacteria in their natural location may allow construction of a 3-dimensional composite heat map that is much richer in landscape-pattern information than that deduced from using a few prelabeled bacterial species. Such composite heat maps may provide pattern recognition for many diseases that derive from microbiota dysbiosis. Second, as the size of the experimental animal increases, spatial resolution of the imaging method drastically increases, making high-resolution imaging of the bacterial landscape in humans feasible. Third, ^18^F-FDS is synthesized by a single-step chemical reaction from an FDA-approved agent (^18^F-FDG), and it has been safely tested in humans as an intravenous infusion (25, 26). FDS is safe (27), and ^18^F is widely used in FDA-approved imaging applications (28). These features, plus the major biodistribution site of FDS being in the gut microbiota, make oral administration of ^18^F-FDS for gut microbiota imaging unlikely to encounter safety issues.

### Conclusion.

^18^F-FDS-based imaging of gut microbiota has the potential to jumpstart a variety of studies that ask whether ecological pattern changes in gut microbiota correlate with particular diseases, diet, chemotherapy, bacterial transplantation, and even behavior. Then, we can ask how to best restore a perturbed microbiota ecology. Such work cannot be performed with currently available methodologies, especially with human and large-animal disease models.

## MATERIALS AND METHODS

### Chemicals and reagents.

Metronidazole, ciprofloxacin, clindamycin, neomycin, and ampicillin were obtained from Sangon Biotech Co., Ltd. (Shanghai, China). Vancomycin was acquired from MSD & Co., Inc. (Hangzhou, China). Luria-Bertani (LB) medium was purchased from Thermo Fisher Scientific (Shanghai, China). Mueller-Hinton (MH) and brain heart infusion (BHI) media were acquired from Becton, Dickinson and Company (Franklin Lakes, NJ, USA). ^18^F-FDG and ^18^F were obtained from the Department of Nuclear Medicine, First Affiliated Hospital of Xiamen University. Staphylococcus aureus RN450 and Escherichia coli BW25113 were from frozen stocks of the Laboratory of Microbial Pathogens, Xiamen University. Probiotics (30 billion CFU/capsule; Island’s Miracle, USA) were purchased from Amazon. Female C57BL/6 mice and male hamsters were purchased from the Beijing Vital River Laboratory Animal Technology Co., Ltd. (Beijing, China), and housed at the Laboratory Animal Center of Xiamen University.

### Preparation of ^18^F-FDS.

^18^F-FDS was prepared as described previously (19) with modification. Briefly, NaBH_4_ (2 mg, 0.053 mmol) was added to a solution of ^18^F-FDG (629 MBq) in 500 μl 0.9% NaCl; the resulting mixture was stirred at 35°C for 30 min. After quenching the reaction with 1 ml acetic acid, pH was adjusted to 7.4 with 1 M HCl, and the mixture was filtered through an Alumina-NSep-Pak cartridge. A single peak was observed via TLC (*R_f_* = 0.99 [80% acetonitrile with 20% water as eluent], *R_f_* = 1.24 for ^18^F-FDG).

### Preparation of 2-^18^F-PABA.

Methods for preparation of 2-^18^F-PABA and reaction intermediates used for 2-^18^F-PABA synthesis are described in Text S1 in the supplemental material as 2-^18^F-PABA was not suitable for gut microbiota imaging and thus used only as a control compound.

### Gut microbiota depletion and bacterial transplantation in mice.

All animal experiments were approved by the Animal Care and Use Committee of the Laboratory Animal Center of Xiamen University (IACUC protocol XMULAC20170367). For continuous antibiotic treatment, animals were allowed to freely drink autoclaved water containing 4 antibiotics (ampicillin at 1 g/liter, metronidazole at 1 g/liter, neomycin at 1 g/liter, and vancomycin at 0.5 g/liter). Drinking water was replaced every 2 days. For antibiotic treatment by oral gavage, animals consumed autoclaved food and water *ad arbitrium*. Ciprofloxacin and clindamycin were administered by oral gavage at a dose of 250 mg/kg of body weight twice daily. The third approach combined the two methods above. To monitor depletion of gut microbiota, fresh fecal samples were homogenized in sterile saline (0.9% NaCl), and CFU was determined using 10-fold serial dilution and plating of samples on LB agar for total culturable aerobic bacteria or on LB agar containing 0.4% glucose and 100 mM sodium nitrate for total culturable anaerobic or facultative anaerobes following incubation in a conventional incubator or in an anaerobic chamber at 37°C for 36 to 48 h.

To reestablish gut microbiota after antibiotic treatment, 3 pieces of fresh fecal samples from untreated mice (one piece from each of 3 mice) were collected and resuspended in 1 ml of sterile saline by vortex mixing; 200 μl of this suspension was administered by oral gavage to gut microbiota-depleted mice twice daily for a week after antibiotic treatment was stopped. For probiotic transplantation, probiotics (Lactobacillus acidophilus, Bifidobacterium bifidum, Bifidobacterium breve, Bifidobacterium infantis, Bifidobacterium lactis, Bifidobacterium longum, Lactobacillus casei subsp. *casei*, Lactobacillus fermentum, Lactobacillus gasseri, Lactobacillus plantarum, Lactobacillus reuteri, Lactobacillus rhamnosus, Lactobacillus salivarius, Streptococcus thermophilus, Bacillus coagulans, Lactobacillus paracasei, Lactobacillus bulgaricus, Lactobacillus helveticus; Island’s Miracle, USA), 10^8^ CFU in 200 μl 0.9% NaCl, were administered by oral gavage after antibiotic treatment was stopped, twice daily for a week. The bacterial titer claimed by the manufacturer was checked by Gram staining and by total viable bacterial count using BHI agar and incubation under both aerobic and anaerobic conditions at 37°C. The Gram stain result was consistent with manufacturer’s titer while the viable count method revealed a 10-fold-smaller bacterial count per capsule. We used our viable count result rather than manufacturer’s titer to calculate the amount of bacterial dose used in probiotic microbiota transplantation.

### PET-CT imaging.

PET-CT imaging was performed using a pinhole collimator PET-CT scanner (Inveon; Siemens, Germany) and standard animal scan procedures. Radionuclide-labeled probe, 200 μCi (7.4 MBq, mouse) or 400 μCi (14.8 MBq, Syrian hamster) in 200 μl or 400 μl 0.9% NaCl, was administered to animals (fasted for 18 h) once by oral gavage. Then, saline was given once per hour until 1 h before imaging to lower residual radiotracer in the upper gastrointestinal (GI) tract. Static PET-CT imaging of gut microbiota-depleted and control mice (*n* = 3 for each group) was performed at 1, 3, 4, and 5 h after oral administration. The animals were anesthetized by 2% isoflurane during PET-CT imaging. The percent injected dose per gram of tissue (%ID/g) derived from PET-CT imaging was used to assess the retention of the radionuclide. All imaging experiments were performed independently with 3 animals.

### Bacterial uptake and retention assays.

For ^18^F-FDS, E. coli (BW25113, LB medium), S. aureus (RN450, MH medium), and C. difficile (VPI 10463, BHI medium) were cultured in liquid medium aerobically (E. coli and S. aureus) or anaerobically (C. difficile) to an optical density at 600 nm of 0.3. Then, 1-ml aliquots of cultures were incubated with 2 μCi (74 kBq) ^18^F-FDS at 37°C for 2 h with rapid agitation. Samples were then sedimented by centrifugation (6,000 × *g*, 5 min), and free radioisotope was removed by washing with saline. Total radioactivity for each sample was measured using an automated gamma counter (Wizard 2480; Perkin-Elmer, Waltham, MA, USA). CFU were enumerated by serial dilution and plating on agar plates and used for radioactivity normalization per 10^6^ CFU. A minimum of three replicates were performed for each assay.

For 2-^18^F-PABA uptake and retention, E. coli (BW25113, LB medium) and S. aureus (RN450, MH medium) were similarly grown and labeled as in ^18^F-FDS uptake assays, with detailed protocols being described in Text S1.

### Biodistribution.

After completion of gut microbiota imaging, ^18^F-FDG- or ^18^F-FDS-treated mice (*n* = 3) were sacrificed. Organs and tissues of interest were dissected and weighed. The radioactivity of the organs and tissues was measured using a gamma counter (Wizard 2480; Perkin-Elmer, Waltham, MA, USA). The biodistribution information of organs and tissues is displayed as the percentage of the injected dose per gram of tissue (%ID/g).

### Statistical analysis.

Quantitative data are presented with error bars as the means ± standard error of the mean (SEM). Statistical differences among groups were determined by Student’s *t* test. Differences were considered significant when *P* values were less than 0.05 (*, *P* < 0.05; **, *P* < 0.01; ***, *P* < 0.001). Coefficient of determination was computed to measure the linear relationship between two quantitative variables.
