# Supplementary figures and images for: In Situ Live Imaging of Gut Microbiota
Source: mSphere. 2021 Sep 29;6(5):e00545-21. doi: 10.1128/mSphere.00545-21 (PMC8550083; doi:10.1128/mSphere.00545-21)

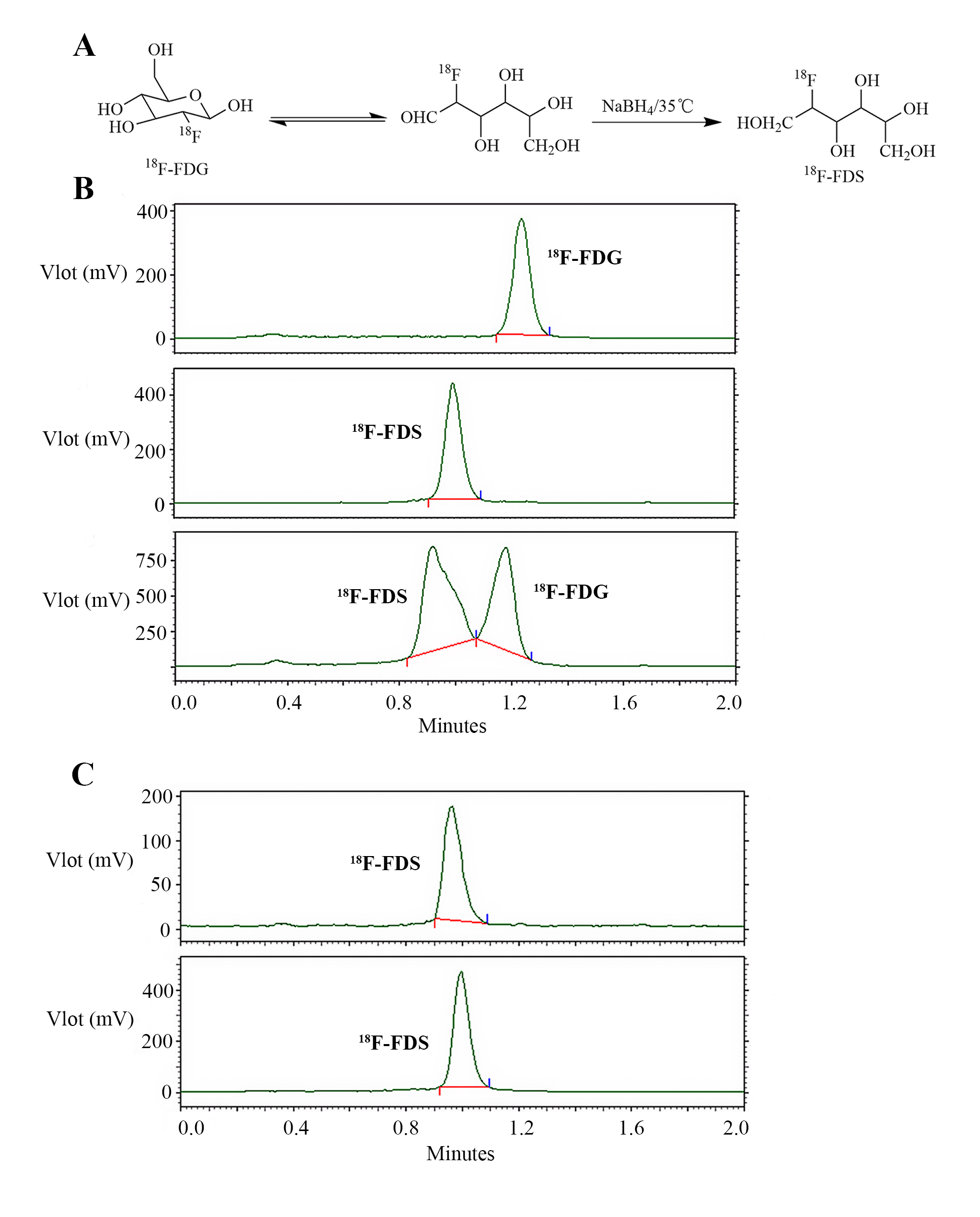

Supplement: FIG S1 [file msphere.00545-21-sf001.tif]

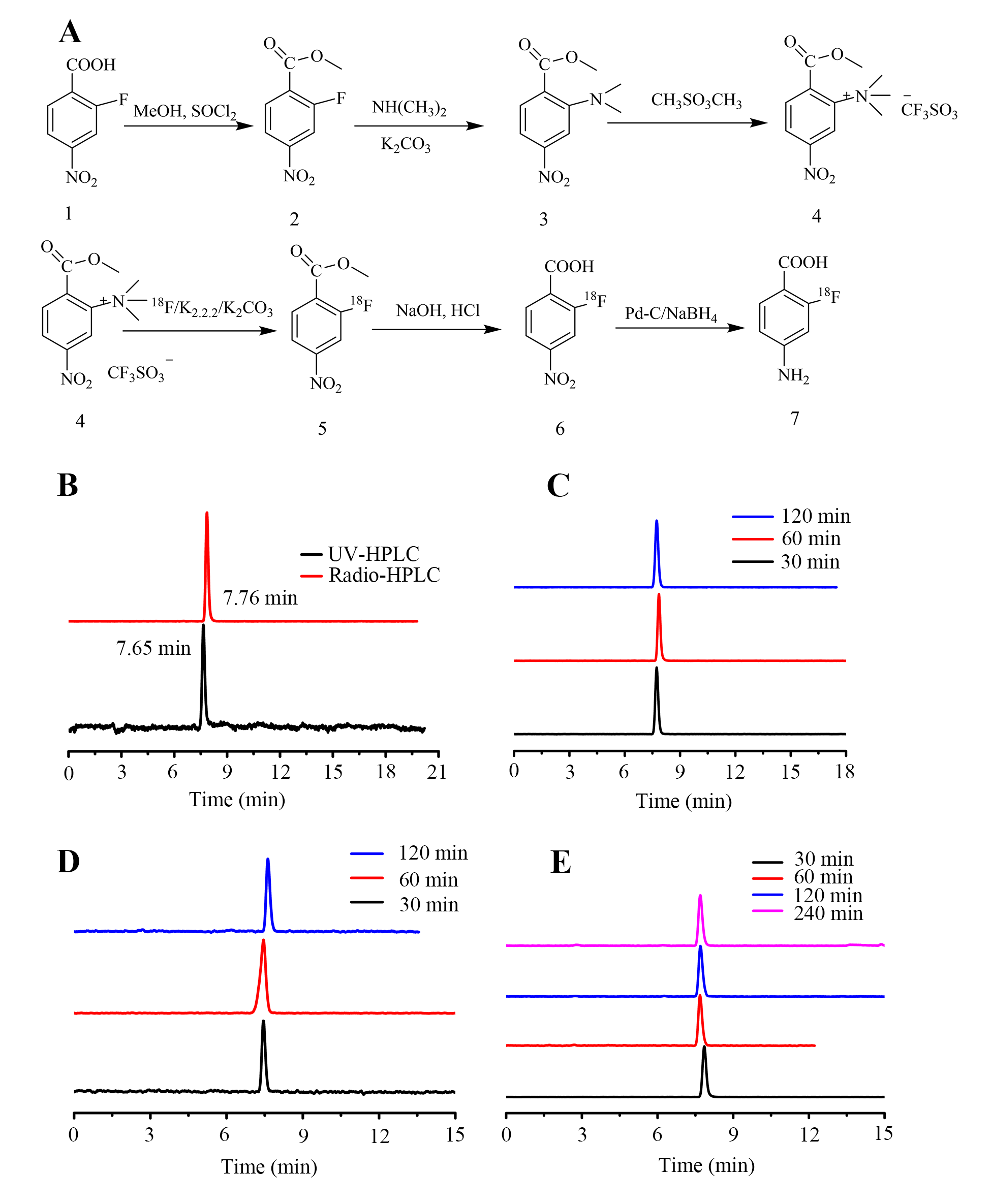

Supplement: FIG S2 [file msphere.00545-21-sf002.tif]

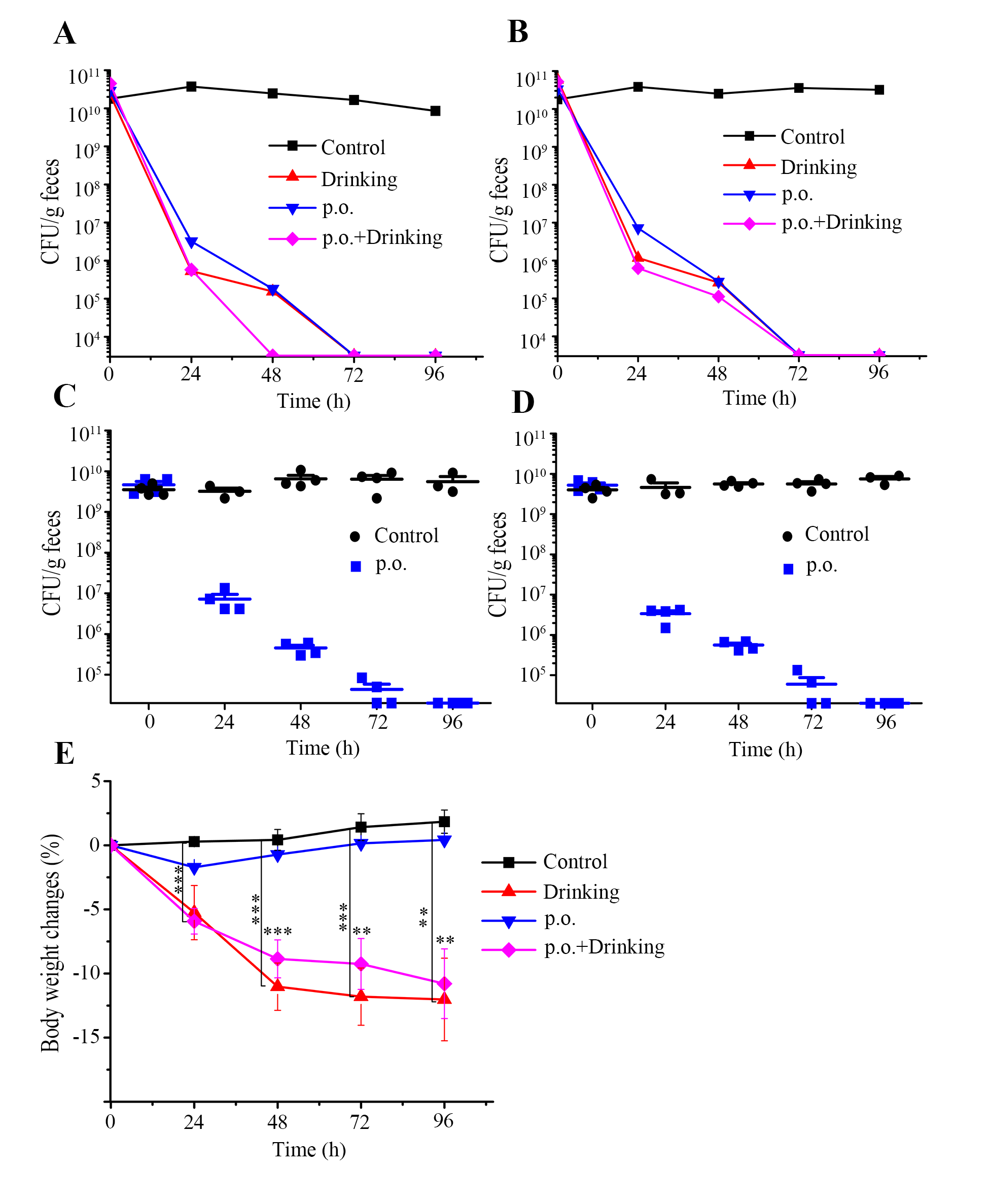

Supplement: FIG S3 [file msphere.00545-21-sf003.tif]

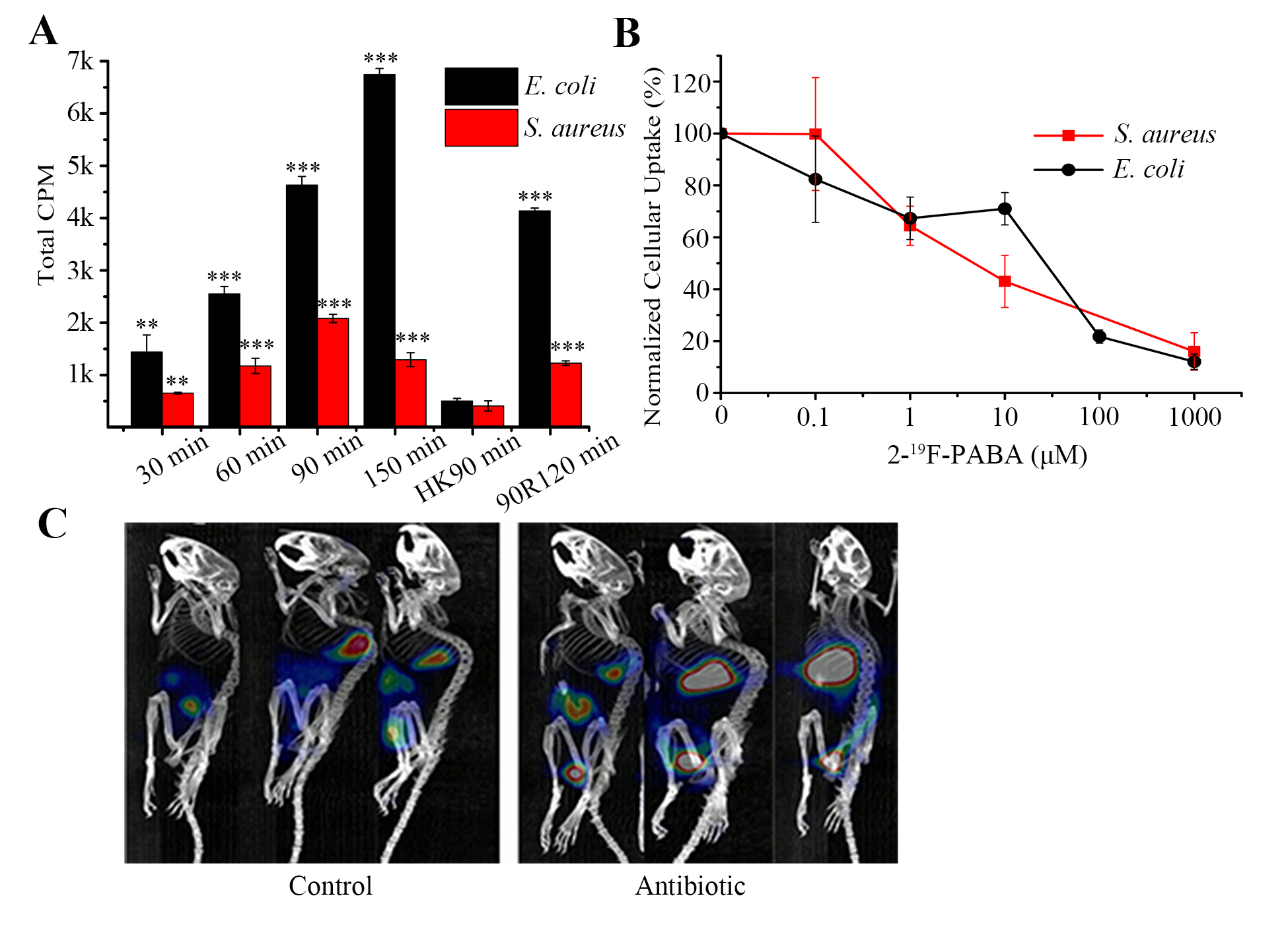

Supplement: FIG S4 [file msphere.00545-21-sf004.tif]

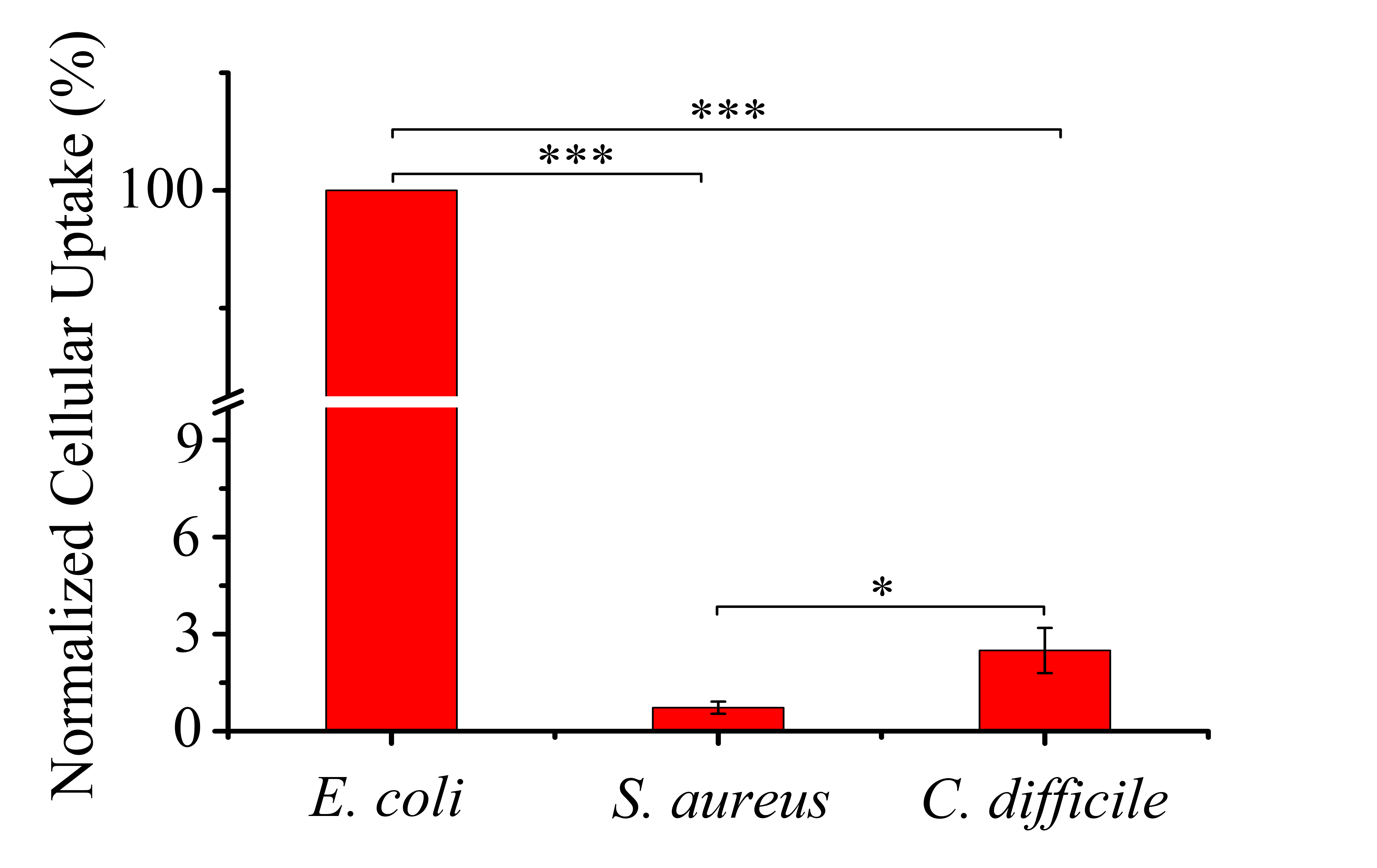

Supplement: FIG S5 [file msphere.00545-21-sf005.tif]
