# Supplementary material for: In Situ Live Imaging of Gut Microbiota
Source: mSphere. 2021 Sep 29;6(5):e00545-21. doi: 10.1128/mSphere.00545-21 (PMC8550083; doi:10.1128/mSphere.00545-21)
Supplement: TEXT S1 [file msphere.00545-21-s0001.docx]

**SUPPLEMENTARY TEXT FOR**

**In situ live imaging of gut microbiota**

Zhi Zhang^a*^, Duo Xu^a,b*^, Jianyang Fang^a,b*^, Dai Wang^a*^, Jie Zeng^a^, Xiaodong Liu^a^, Shouqiang Hong^a^, Yunxin Xue^a^, Xianzhong Zhang^a,b#^, Xilin Zhao^a,c,d#^

^a^State Key Laboratory of Molecular Vaccinology and Molecular Diagnostics, School of Public Health, Xiamen University, South Xiang-An Road, Xiang-An District, Xiamen, Fujian Province 361102, China.

^b^Center for Molecular Imaging and Translational Medicine, School of Public Health, Xiamen University, Xiamen 361102, China.

^c^Public Health Research Institute, New Jersey Medical School, Rutgers University, 225 Warren Street, Newark, NJ 07103, USA.

^d^Department of Microbiology, Biochemistry & Molecular Genetics, New Jersey Medical School, Rutgers University, 225 Warren Street, Newark, NJ 07103, USA.

**1. Optimization of antimicrobial treatment regimens for depletion of murine gut microbiota**

Before imaging murine gut microbiota, we performed a control experiment in which the gut was depleted of bacteria by antibiotic treatment. We compared the effects of three antimicrobial combinations on the total culturable gut microbiota total culturable fecal microorganisms was lowered by about five orders of magnitude following a) addition of a combination of ampicillin, neomycin, metronidazole, and vancomycin to drinking water, b) administration of clindamycin and ciprofloxacin by oral gavage, or c) by combination of procedures a) and b). On the first day of antibiotic treatment, the total number of bacteria culturable under either aerobic or anaerobic conditions decreased by 3-4 orders of magnitude. By the fourth day, the culturable microorganisms in all three protocols decreased below the detection limit (6 orders of magnitude; Fig. S3A-D).

When we measured body weight changes of mice during daily administration of antibiotics, the combination of four antibiotics in drinking water and the drinking water plus the oral regimen combination led to severe weight loss, consistent with previous studies of dehydration symptoms ([1](#_ENREF_1)) (Fig. S3E). The oral regimen of only two antibiotics, clindamycin plus ciprofloxacin, showed no weight loss. Thus, all three regimens significantly deplete gut microbiota in mice, but only oral administration of ciprofloxacin plus clindamycin had little adverse effect (Fig. S3E). Subsequent experiments used the two-drug oral gavage regimen to deplete gut microbiota.

**2. Synthesis and characterization of 2-[^18^F]fluoro-para-aminobenzoic acid (2-^18^F-PABA)**

The synthetic methods used for preparing 2-^18^F-PABA and intermediate compounds used for its synthesis are described below.

**Preparation of methyl -2-fluoro-4-nitrobenzoate**

Thionyl chloride (5.0 mmol, 0.36 mL) was added dropwise to a vial containing 2 mL methanol on ice. 2-fluoro-4-nitrobenzoic acid (555 mg 3 mmol) in methanol was then added, and the mixture was stirred at ambient temperature for 4 h. The solvent was removed by evaporation. The crude residue was purified by silica chromatography to give methyl-2-fluoro-4-nitrobenzoate (500 mg, 83.7% yield). All compounds synthesized were authenticated by ^1^H NMR.

**Preparation of methyl 2-(dimethylamino)-4-nitrobenzoate**

Potassium carbonate (600 mg, 4.3 mmol) and dimethylamine hydrochloride (195 mg, 2.4 mmol) were added to a solution of methyl-2-fluoro-4-nitrobenzoate (400 mg, 2 mmol) in DMSO (5 mL). The suspension was stirred under an argon atmosphere for 10 h at 55 °C. The reaction mixture was concentrated, and the residue was dissolved in dichloromethane (10 mL) and washed with water (2×5 mL), brine dried over Na_2_SO_4_, and concentrated in vacuo. This crude product was purified using silica gel column chromatography to yield the desired compound, methyl-2-(dimethylamino)-4-nitrobenzoate (270 mg, 60% yield).

**Preparation of methyl 2-(trimethyl ammoniumtriflate) 4-nitrobenzoate**

Methyl trifluoromethanesulfonate (492 mg, 3 mmol) was added dropwise to a stirred solution of methyl-2-(dimethylamino)-4-nitrobenzoate (224 mg, 1 mmol) in anhydrous dichloromethane (70 mL) under a nitrogen atmosphere. A white precipitate appeared after stirring for 24 h at room temperature; then diethyl ether was added into the mixture. The product precipitated, and the solvent was decanted. The solid was washed with ethyl acetate（5 x 3 mL without further purification to give the desired compound (182 mg, 48.6% yield).

**Preparation of 2-^18^F-4-aminobenzoic acid (2-^18^F-PABA)**

The radiotracer 2-^18^F-PABA was prepared using a two-step procedure. First, methyl-2-(trimethyl ammoniumtriflate)-4-nitrobenzoate (1 mg) in DMSO (250 µL) was added to a vial containing dried K^18^F/Kryptofix 2.2.2/K_2_CO_3_ . The vessel was sealed and heated at 90 °C for 10 min. NaOH (0.5 N, 1.5 mL) was added, and the resulting mixture was heated to 115 °C for 10 min. The crude product was diluted with water (10 mL) and 5 N HCl (0.3 mL). The solution was passed through a C18 Sep-Pak cartridge (Waters, Milford, Massachusetts, USA­). The product, trapped on the cartridge, was washed with water (10 mL) and dried with air. The 2-[^18^F]F-4-nitrobenzoic acid product was eluted with methanol (1.5 mL). Second, the above solution of 2-[^18^F]F-4-nitrobenzoic acid in methanol was transferred to a flat-bottom glass vial containing NaBH_4_ (10 mg) and 10 wt % Pd-C (4 mg). Reduction proceeded for 7 min at room temperature before quenching with 100 µL of 5 N HCl. NaOH (5N) was added to adjust pH to 4-5. The solution was filtered and purified via HPLC. The HPLC column was eluted with 0.1% trifluoroacetic acid-water (solvent A) and methanol (solvent B) at a flow rate of 1.0 mL/min with the following gradient: 0–10 min: 80% A, 10.01–16 min: 80% B, 16.01–25 min: 20–80% A, 25.1-30 min: 80% A. The product was collected, and the solvent was evaporated by rotary evaporation. The 2-^18^F-PABA product was re-dissolved in 5% ethanol and filtered through a 0.22-µm Millipore filter before use.

**Bacterial uptake and retention assays**

*E. coli* (BW25113, LB medium) and *S. aureus* (RN450, MH medium) were grown aerobically to absorbance at 600 nm = 0.3. 2-^18^F-PABA uptake assays were performed by co-incubating bacterial cultures with 5 µCi (185KBq)/mL at 37˚C with rapid agitation until the desired time point. At 90 min after 2-^18^F-PABA addition, samples were sedimented by centrifugation, and free radioisotope was removed, after which the pelleted bacterial samples were suspended in 37˚C pre-warmed fresh medium for incubation for 2 more h to assess bacterial retention of radiotracer. As a negative control, heat-killed (90˚C for 30 min) bacteria were similarly incubated with 2-^18^F-PABA. For competition experiments, bacterial cultures were incubated for 2 h at 37˚C with increasing concentrations of cold PABA probe from 0 to 1,000 µM along with 5 µCi (185KBq)/mL 2-^18^F-PABA. Bacteria were collected by centrifugation and washed three times with saline; total radioactivity for each sample was measured using an automated gamma counter (WIZARD 2480, Perkin-Elmer, Waltham, MA, USA). CFUs were enumerated by serial dilution and plating on agar plates. A minimum of three replicates were included for each assay.

In vitro characterization of 2-^18^F-PABA was performed before this compound was used as a radio tracer for gut microbiota imaging. Uptake of 2-^18^F-PABA by cultured *S. aureus* and *E. coli* was observed, with uptake by *E. coli* being 2-4 times greater than by *S. aureus* (Fig. S4A); little uptake was observed with heat-killed bacteria. The radioactivity signal was retained for 120 min after removal of 2-^18^F-PABA from the medium (Fig. S4A). As expected, uptake of 2-^18^F-PABA was inhibited by adding non-radioactive PABA to bacterial cultures (Fig. S4B).

**3. Supplementary References**

1. Hill DA, Hoffmann C, Abt MC, Du Y, Kobuley D, Kirn TJ, Bushman FD, Artis D. 2010. Metagenomic analyses reveal antibiotic-induced temporal and spatial changes in intestinal microbiota with associated alterations in immune cell homeostasis. Mucosal Immunol 3:148-58.
